# Evaluation of complete functional status of patients with stroke by Functional Independence Measure scale on admission, discharge, and six months poststroke

**Published:** 2016-10-07

**Authors:** Seyed Mansoor Rayegani, Seyed Ahmad Raeissadat, Ebrahim Alikhani, Masume Bayat, Mohammad Hasan Bahrami, Afshin Karimzadeh

**Affiliations:** ^1^ Physical Medicine and Rehabilitation Research Center, Shahid Beheshti University of Medical Sciences, Tehran, Iran

**Keywords:** Stroke, Function, Disability Evaluation

## Abstract

**Background:** To evaluate the patients with stroke by Functional Independence Measure (FIM) scale, at the times of admission to hospital, discharge, and six-month poststroke, and to determine the level of improvement in patients after rehabilitative procedures.

**Methods:** A total number of 108 patients with stroke entered the study who were admitted to neurology ward. They all received rehabilitation consultation, and occupational and physical therapies were prescribed for them. Finally, their functional status was evaluated by FIM scale.

**Results:** The median (and range) of FIM scores were 86 (15-119), 102 (16-123) and 119 (17-126) at admission, discharge, and after six-month follow-up, respectively. Our observations showed a significant improvement in FIM scores (P < 0.001). About 13, 30, and 76 percent of the patients in individual functional tasks of motor domain and 61, 75, and 86 percent in cognitive domain got the score of 6 or 7 (complete or partial independence) on admission, discharge, and after six months, respectively. There was a reverse correlation between age and FIM improvement and also duration of hospitalization (P = 0.002).

**Conclusion:** The study showed that the FIM is a valid tool for evaluation of patients with stroke, their follow-up and tracking the disease course. Moreover, we concluded that patients with stroke make a significant improvement in their functional status overtime. The exact effect of rehabilitative procedures and comparison with no treatment, must be assessed in separate studies.

## Introduction

Cerebrovascular accident (CVA) is the third most common cause of death worldwide, and one of the most common causes of disability in elderly population.^1^^,^^2^ About 15 to 30 percent of patients with stroke suffer persistent disabilities, and only 13 percent of affected subjects return to work.^3^^,^^4^ Stroke can impact different aspects of subject’s life, including gross and fine motor control, mobility, activities of daily living (ADL), mood, speech, comprehension and cognition.

Depending on the involved artery, the size and location of supplied cerebral area and the extent of resulted brain damage, patients might experience various complications. Postural disorders, sensory and motor deficits, hemiplegia or hemiparesis, cognition and comprehension difficulties, memory impairment, decreased self-care and ADL abilities,^5^ emotional and mood disorders,^6^ sexual dysfunction,^7^ and decreased social participation are some typical consequences of stroke. These complications directly affect subject’s role fulfillment, and finally lead to decreased patient’s quality of life.^8^ Functional impairment is a common long lasting sequel of central nervous system (CNS) disorders which can cause patient disability.

One important aspect of clinical research is selecting appropriate measurement tools to quantify the clinical consequences of diseases, effectiveness of applied treatments, and comparison of the results.^9^ Since most parameters in rehabilitation field are somehow qualitative rather than quantitative, this requirement is even more prominent.

Planning an appropriate rehabilitation program for stroke patients needs careful and comprehensive assessment of the subjects and their physical and functional condition. Wide range and chronic nature of CVA complications make this necessary to evaluate several aspects of such patients’ life, including disability, functional impairment, and quality of life. It is important to consider not only short term, but also long lasting consequences. This can provide the health care system with useful information for planning the rehabilitation protocols.^10^

One useful way to estimate the level of functional independence in CVA patients is evaluation of ADL. A valid tool in this field is Functional Independence Measure (FIM).^4^^,^^11^ FIM questionnaire was first introduced in 1983. It was presented by American Congress of Rehabilitation Medicine and American Academy of Physical Medicine and Rehabilitation as a promotion of Barthle Index. It is a tool for collection and comparison of rehabilitation outcomes, measurement of patients’ progress, and planning treatment protocols. The producers planned it for more precise evaluation of patients’ functional status, at different stages of disease.^4^^,^^11^ ADL, which are the purpose of this test include: self-care, eating, grooming, bathing, dressing, toileting, swallowing, sphincter control, mobility, transfer, and locomotion. It does not include home management activities.

The scale contains 18 items, of which 13 items are in physical domains and 5 items are related to cognition. Motor items measure self-care, sphincter control, locomotion, and transfer. Cognitive ones evaluate subject’s communication and social cognition. Based on level of independence, each item is scored from 1 to 7, where 1 indicates total dependence and 7 represents complete independence. Possible scores range from 18 to 126. Obtaining higher score means more independence in ADL FIM score is indicative of patients’ level of disability and the burden of their care.^1^^,^^4^^,^^11^

Subjects are routinely evaluated by FIM questionnaire on admission and discharge from rehabilitation setting.^4^^,^^12^^,^^13^ The questionnaire is easy to apply, and takes a fairly short time to be completed (about 30 minutes for answering questions and 10 minutes for final scoring).

The FIM score has not been applied to Iranian population yet, also the validity and reliability of Persian translation is needed to be approved. The purpose of this study was to apply this tool to assess independence level in Iranian patients with stroke. This study can facilitate more extended use of this scale in rehabilitation settings.

## Materials and Methods

It was a descriptive observational study. A total number of 108 patients with stroke took part in the study, in whom the stroke diagnosis (based on clinical and imaging exams) was finally confirmed by a neurologist. Subjects were randomly chosen from stroke patients of General Neurology Ward of Shohadaye Tajrish Hospital, Tehran, Iran, in the time interval between January to September 2012. The inclusion criteria were as follows:

1. It was the first stroke attack.
2. Stroke led to functional impairment.
3. Patients were medically and hemodynamically stable.
4. At least one day was passed from the accident.
5. Patients did not take neuroprotective agents.

Exclusion criteria included:

1. History of previous stroke.
2. Evidence of transient ischemic attack or subarachnoid hemorrhage.
3. History of orthopedic surgeries, malignancy or neurological disorders such as Alzheimer’s disease.
4. Any other condition with the potency of causing disability and functional impairment.
5. Patient unwilling to join the study.

After entering the study, patients’ medical characteristics, including gender, age, type of stroke (ischemic, hemorrhagic), duration of hospital admission, and their FIM scores, were all documented. Subjects’ functional status was assessed using FIM questionnaire on admission, discharge, and six months after the incidence of stroke. The evaluations were accomplished by a physical medicine and rehabilitation specialist. On admission and discharge visits, patient were directly observed by a physician, during the task fulfillment, and the level of independence was detected. Since the true bathing situation was not accessible in clinic, we made an exception about this item and asked it orally. In six-month follow-up visit, we made telephone calls and questioned the patients about their condition, and collected data based on their verbal answers.

In preliminary visit, after explanation of study process, a written consent was obtained from all eligible patients. Each assessment session took about 30 minutes on average, and all visits were accomplished by the same physician.

Patients were admitted by neurology service and referred to rehabilitation specialist by their neurologist. All patients received physical or occupational therapy, during hospital admission. They were also ordered physical and occupational therapies by rehabilitation specialist for the period after discharge, but the rehabilitation program did not happen in a systematic inpatient or outpatient rehabilitation setting.

Data analysis was performed using SPSS software (version 18, SPSS Inc., Chicago, IL, USA). Mean and standard deviation (SD), or median and range (minimum-maximum) were used to present the quantitative scales. For qualitative scales, we obtained the distribution frequency statistics. In order to detect the relations between qualitative scales, the chai-square test was used. We applied Spearman’s rank correlation test to find the linear correlation between two quantitative scales. In order to detect the dependence between changes in quantitative and qualitative scales, the Student’s t-test or Mann-Whitney U test was performed. If the qualitative scale had more than two conditions, the one-way analysis of variance test (Kruskal-Wallis) was applied. We used LSD (least square deviation) for post-hoc test. Analysis of changes in each subject’s FIM score was accomplished by the Wilcoxon test. Friedman test was also performed to detect the meaningful changes in FIM scores. For predicting the death probability, patients’ characteristics were analyzed by backward multiple logistic regression test.

## Results

A total number of 108 eligible patients entered the program with documented diagnosis of stroke. All patients were visited by an expert neurologist and the diagnosis of CVA was based on their clinical and imaging findings. In later stages, 29 patients died (14 patients during the hospital admission and 15 after discharge). Finally, about 73% of the patients (79 subjects) completed the follow-up visits and their data was analyzed. Patients’ characteristics including age, gender, stroke type, and duration of hospitalization are listed in table 1.

**Table 1:** Demographic characteristics

**Variable**		**Patients entered ** **the study**	**Patients died**	**Patients survived to ** **final stage**	**P** *
Number of patients		108 (100)	29 (27)	79 (73)	-
Age**		62 ± 17, (16-91)	73 ± 17, (26-91)	58 ± 16, (16-89)	< 0.001
Age group (year)	< 40	13 (12)	2 (7)	11 (14)	0.002
	40-60	39 (36)	4 (14)	35 (44)	
	> 60	56 (52)	23 (79)	33 (42)	
Gender	Male	70 (65)	19 (66)	51 (65)	0.926
	Female	38 (35)	10 (34)	28 (35)	
Stroke type	Embolic	20 (18)	3 (10)	17 (21)	0.002
	Thrombotic	59 (55)	11 (38)	48 (61)	
	ICH	29 (27)	15 (52)	14 (18)	
Days stayed at hospital***		8 (2-92)	12 (3-92)	6 (2-63)	< 0.001

* P-values determine the significant differences between dead and alive patients;

** Mean ± SD (Standard deviation), (minimum-maximum);

*** Median (minimum-maximum); ICH: Intracerebral hemorrhage

**Figure 1:**
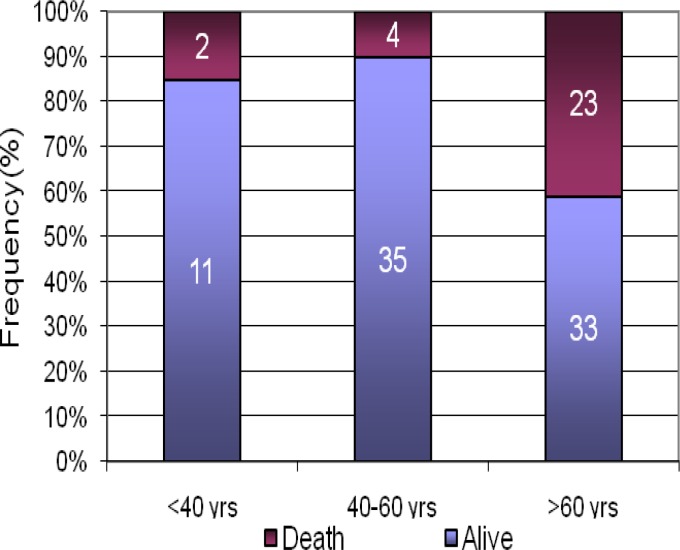
Death frequency among different age groups

A higher age was found in expired patients group (P < 0.001). Moreover, the mean FIM score of these subjects was lower, at both admission and discharge visits (P < 0.001). Figure 1 shows the distribution of death rate in different age groups.

Our findings showed that the incidence of death in subjects older than 60 was 11 times and 22 times higher than below 40 and 40-60, respectively. Beside age, the FIM score had an independent effect on the survival rate. It was shown that 10 score increase in initial FIM score resulted in 1.55 times increase in subject’s survival rate. Median, minimum and maximum of FIM scores are shown in figure 2.

Table 2 shows the scores of motor (maximum: 91) and cognition (maximum: 35) domains on follow-up visits.

As it is evident, the scores of all categories improved over time (P < 0.001). It is noteworthy that about 13, 30, and 76 percent of subjects achieved the level of independence (score 6 or 7) in individual functional tasks of motor domain (such as self-care and mobility activities), at the times of admission, discharge, and six-months poststroke, respectively. Also, in cognitive domain, about 61, 75, and 86 percent were considered independent at the times of admission, discharge, and six-month follow-up, respectively.

**Figure ‎2:**
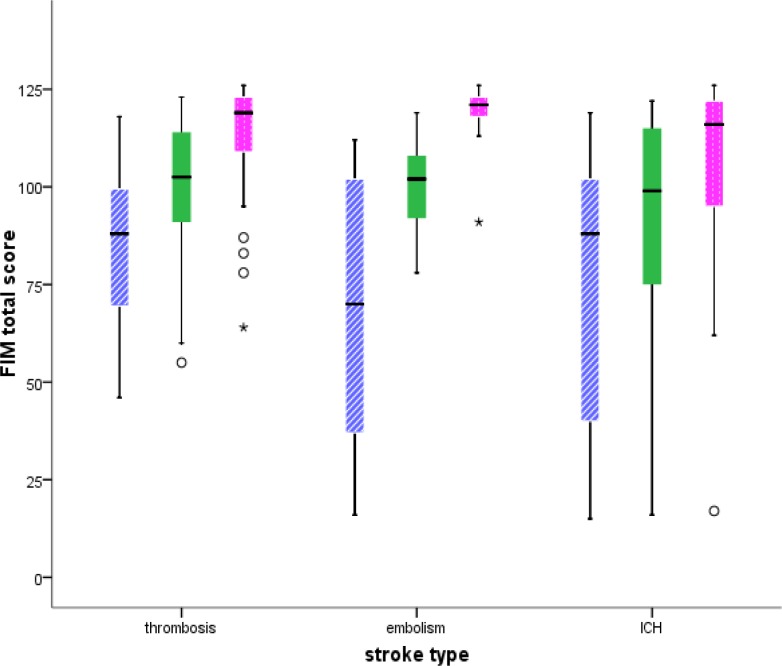
Total Functional Independence Measure (FIM) score distribution at the time of admission, discharge, and six-month follow-up

The study showed that there was a direct linear correlation between each subject’s total FIM score and the scores of motor and cognition domains on all follow-up visits. It means that, obtaining a higher score on initial evaluation led to higher scores at the times of discharge and six months later. Also, more independence in motor domain accompanied better cognitive function.

There was a significant inverse relation between subjects’ age and their FIM scores, at the times of discharge and six-month follow-up visit (P < 0.001). However, this association was not obtained between age and admission’s FIM score. We also observed that the gain in FIM score was inversely linked to the age and it was significantly lower after 60 years old (P = 0.010). We did not observe any association between gender and FIM scores. No significant correlation was observed between type of CVA and FIM scores (Table 3).

**Table 2:** Patients functional status according to Functional Independence Measure (FIM) score

**FIM scale**	**Admission**	**Discharge**	**Six-month follow-up**	**P**
Motor domain score	53 (12-86)	72 (12-88)	86 (12-94)	< 0.001
Cognition domain score	32 (5-35)	33 (5-35)	33 (5-35)	< 0.001
Self-care	23 (5-42)	32 (5-42)	40 (5-42)	< 0.001
Sphincter control	14 (2-14)	14 (2-14)	14 (2-14)	< 0.001
Locomotion	11 (3-20)	14 (3-21)	19 (3-21)	< 0.001
Transfer	7 (2-14)	9 (2-14)	12 (2-18)	< 0.001
Communication	11 (2-14)	13 (2-14)	14 (2-14)	< 0.001
Social cognition	21 (3-21)	21 (3-21)	21 (3-21)	< 0.001

FIM: Functional Independence Measure

**Table 3:** Correlation coefficient between patients’ Functional Independence Measure (FIM) score and other characteristics

		**Age**	**Days of stay ** **at hospital**	**Total FIM score on**		
				**Admission**	**Discharge**	**Six months**
Days of stay at hospital		-0.16 (0.17)	-	-	-	-
Total	Admission	0.07 (0.56)	-0.43 (< 0.01)	-	-	-
FIM	Discharge	-0.19 (0.10)	-0.46 (< 0.01)	0.85 (< 0.01)	-	-
score at	Six months	-0.33 (< 0.01)	-0.33 (< 0.01)	0.53 (< 0.01)	0.79 (< 0.01)	-

The study showed that longer duration of stay at hospital was associated with lower scores of total FIM, and also motor and cognition subscales, on discharge and six-month follow-up visits (Table 2).

The gain in subjects’ FIM scores was inversely correlated to the admission FIM score. So subjects with less initial FIM score, showed more remarkable improvement in latter stages. This relation was also observed in motor and cognition subscales (r = -0.66, r = -0.86, and r = -0.63) (Table 2).

## Discussion

We observed a significant improvement in total FIM score and also its motor and cognition subscales on follow-up visits. The mean (and range) of total FIM scores were 86 (15-119), 102 (16-123) and 119 (17-126) for admission, discharge and six-month poststroke, respectively. According to some studies, FIM score of more than 108 is roughly indicative of home independence.^14^ In our study, subjects achieved this level of independence on six-month follow-up visits.

Based on the study by Beninato et al.^15^, the least significant changes for total FIM, motor and cognition scores were 22, 17 and 3, respectively. Compared to their findings our patients showed these least significant changes. Our findings revealed a direct linear correlation between scores of total FIM and its motor and cognition subscales at all follow-up sessions. Considering this, higher FIM score on admission led to higher scores on discharge and six months afterward. These results conformed other studies’ findings.^8^^,^^16^^-^^18^

According to our study, gains in motor domain were significantly higher than cognition. This improvement was specifically observed in category of self-care. Assessment of results revealed a mean increase of 12 (range: 6-80) and 13 (range: 1-40) in total FIM scores at admission and discharge, respectively. Comparison of these findings showed no significant difference. The above statement applied to motor domain too. It was different in cognition domain. The results showed significantly higher improvement in subjects’ cognition domain of FIM score during the hospital admission. We observed fairly small changes in patients’ cognitive status at staying home periods. On the other hand, about 86% of subjects got the maximum score of cognition domain on six-month follow-up visits. Putting these findings together, it can imply the relative weakness of FIM score in detecting subtle changes of cognitive status. This conclusion is consistent with the results of Hall et al. study.^16^ Another study by Tokunaga et al.^19^ showed that the gain in FIM score, in completely dependent or completely independent subjects is minimal. According to this, not much of improvement is expected in functional status of these patients.

In our study, the overtime improvement in FIM score was inversely linked to subject’s age. The gain in total FIM score and motor domain were significantly lower in those aged over 60. Our findings match the observation of other trials. Studies confirm that older subjects gain lower FIM scores.^17^^,^^20^^,^^21^ Tur et al.^20^ found that in addition to age, the time gap between accident and admission to hospital and also initial FIM score are predictive of FIM score at discharge. In our study, the age and FIM score of preliminary visit along with duration of hospital residence significantly affected the FIM score at discharge but age did not have a significant effect on admission and the FIM score at discharge. The FIM score of six-month follow-up visit was significantly affected by age, but there was a rather weak correlation coefficient (-0.33). Tokunaga et al.^19^ showed in their study that as the age increased, the admission FIM score and its improvement significantly decrease.

One final objective of stroke rehabilitation is release of patients to home. According to the study by Koyama et al.,^22^ higher age and lower FIM scores decrease the possibility of subject’s discharge to home. They also showed that the age and initial FIM were inversely correlated with FIM improvement. These observations are compatible with ours. In our patients, the FIM scores of discharge and six-month follow- ups were inversely correlated with the admission FIM score. This dependence was also observed in motor and cognition domains. Also, assessment of expired patients’ characteristics revealed that the age and initial FIM score were good predictors of death. In our study, patient’s gender and type of stroke did not affect the FIM scores at admission or follow-up visits. We could not find any study evaluating these two factors.

Our survey on the studies of FIM score showed that it has been used as a measurement device in rehabilitation of stroke, traumatic brain or spinal cord injury, and multiple sclerosis (MS), in elderly and youth populations.^23^^,^^24^ In a systematic review by Chumney and colleagues^4^ in 2010 for validation of FIM questionnaire in stroke patients, they observed that despite its limitations, the FIM score can accurately predict the stroke outcomes. Hamilton et al.^25^ evaluated interrater reliability of FIM score, and its motor and cognition domains and also, FIM item score agreement in 1081 patients. Intraclass correlation coefficients (ICC) for total FIM, motor and cognition domain were 0.96, 0.96, and 0.91, respectively. ICC for subscales score ranged from 0.89 (social cognition) to 0.94 (self-care). They concluded that the FIM, when used by trained rehabilitation clinicians, is reliable enough. In study by Kwon et al.,^26^ a high correlation was observed between motor component of FIM, Barthel index and Modified Ranking Scale. In another study by Dodds et al.,^27^ Uniform Data System (UDS) data on 11102 general rehabilitation inpatients were examined and they conclude that the FIM has high internal consistency. Also, Hall et al.^28^ reported a high correlation coefficient between FIM and disability rating scale.

One shortcoming of FIM scale is that this tool evaluates subject’s independence in ADL performance, but not the way of its accomplishment. Many neurobehavioral disorders affect the quality of task performance, but not just the happening of it. In such a case, despite final task accomplishment, the occurrence of several errors place obstacles in the patient’s way to meet the purpose. Elimination of this defect needs applying more sophisticated and precise measurement tools.

Finally, it is noteworthy that FIM score must be applied by a skilled practitioner. It is self-evident that lack of enough training might threat the measurement reliability. We accomplished the six months follow-up evaluation through a telephone call. There are some studies that used telephone interview for evaluation of subjects’ FIM scale.^29^ Also in a study by Smith et al.,^30^ it was shown that there is good intermodal agreement for telephone assessment using the FIM and in-person assessment. They also demonstrated that the main factor affecting the data collection was subject’s communication skills.^30^ Despite these, it was better using the version available for telephone interviews and this can be considered as a limitation of our study. The other potential source of bias in current study was relatively wide range of discharge visits. According to our findings, time had a positive effect on patients’ FIM scale. Although it was not as wide as some other studies,^31^ this difference in timing of follow-up visit might have affected the results.

In current study, we designed the rehabilitative plans subjectively and based on patients’ individual needs and deficits. According to our knowledge, a predetermined standard CVA rehabilitation protocol has not been defined yet. Assessment of different rehabilitation plans and comparison of their effects needs to be accomplished in separate clinical trials with precise planning of different rehabilitation protocols. We suggest planning more clinical trials to evaluate the effect of different rehabilitation options.

## Conclusion

The study showed that the FIM is a valid tool for evaluation of stroke patients, their follow-up, and tracking the disease course. Also, we concluded that stroke patients make a significant improvement in their functional status overtime. The exact effect of rehabilitative procedures and comparison with no treatment must be assessed in separate studies.

## Notes:

**How to cite this article: **Rayegani SM, Raeissadat SA, Alikhani E, Bayat M, Bahrami MH, Karimzadeh A. Evaluation of complete functional status of patients with stroke by Functional Independence Measure scale on admission, discharge, and six months poststroke. Iran J Neurol 2016; 15(4): 202-8.
